# Use of electronic health records from a statewide health information exchange to support public health surveillance of diabetes and hypertension

**DOI:** 10.1186/s12889-019-7367-z

**Published:** 2019-08-14

**Authors:** Roberta Z. Horth, Shelly Wagstaff, Theron Jeppson, Vishal Patel, Jefferson McClellan, Nicole Bissonette, Michael Friedrichs, Angela C. Dunn

**Affiliations:** 1 Epidemic Intelligence Service, Division of Scientific Education and Professional Development, CDC, Atlanta, Georgia USA; 2 Utah Department of Health, Salt Lake City, UT 84114 USA; 3 Utah Health Information Network, Murray, UT USA

**Keywords:** Health information exchange, Electronic health records, Chronic disease epidemiology, Diabetes, Hypertension, Utah epidemiology, Public health surveillance, Health informatics, Prevalence

## Abstract

**Background:**

Electronic health record (EHR) data, collected primarily for individual patient care and billing purposes, compiled in health information exchanges (HIEs) may have a secondary use for population health surveillance of noncommunicable diseases. However, data compilation across fragmented data sources into HIEs presents potential barriers and quality of data is unknown.

**Methods:**

We compared 2015 patient data from a mid-size health system (Database A) to data from System A patients in the Utah HIE (Database B). We calculated concordance of structured data (sex and age) and unstructured data (blood pressure reading and A1C). We estimated adjusted hypertension and diabetes prevalence in each database and compared these across age groups.

**Results:**

Matching resulted in 72,356 unique patients. Concordance between Database A and Database B exceeded 99% for sex and age, but was 89% for A1C results and 54% for blood pressure readings. Sensitivity, using Database A as the standard, was 57% for hypertension and 55% for diabetes. Age and sex adjusted prevalence of diabetes (8.4% vs 5.8%, Database A and B, respectively) and hypertension (14.5% vs 11.6%, respectively) differed, but this difference was consistent with parallel slopes in prevalence over age groups in both databases.

**Conclusions:**

We identified several gaps in the use of HIE data for surveillance of diabetes and hypertension. High concordance of structured data demonstrate some promise in HIEs capacity to capture patient data. Improving HIE data quality through increased use of structured variables may help make HIE data useful for population health surveillance in places with fragmented EHR systems.

**Electronic supplementary material:**

The online version of this article (10.1186/s12889-019-7367-z) contains supplementary material, which is available to authorized users.

## Background

The global burden of chronic, non-communicable diseases, such as diabetes and hypertension, exceeds that of communicable diseases in most countries; yet, few countries mandate reporting of this data. Public health agencies in many countries, including in the United States, rely on data from population-level surveys for surveillance of diabetes and hypertension [1, 2]. Although surveys provide useful information on the health of populations, they are cross-sectional and often rely on self-reported clinical measures. Electronic health records (EHR) have the potential to complement these surveys by providing near real-time, longitudinal data, allowing disease monitoring of persons over time.

Healthcare organizations worldwide have been rapidly adopting EHR systems, and national EHR’s are now reported in 47% of countries [3]. These hundreds of different EHR systems in use across countries, capture data in different formats, and are often lack interoperability. The United States has a highly fragmented healthcare system with a diversity of EHR systems; in 2016, 78% of office-based physicians were using a certified EHR system [4]. In places with fragmented healthcare systems, health information exchanges (HIEs) can function to collect EHR data across healthcare networks and provider types into one interoperable repository. Though the primary function of HIEs is to facilitate access to patient information for clinical care, data collected by HIEs can potentially have a secondary function in public health to monitor disease and quantify burden at the population-level [5].

The broad geographic and demographic reach of HIEs make it a potentially rich resource for disease surveillance. Small area monitoring of chronic disease patterns with adequate precision longitudinally would be possible with an HIE-based surveillance system. Yet, the compilation of data across multiple data sources into an HIE presents potential for data quality issues, compounding quality issues inherent to each different EHR system. Studies documenting these quality issues and evaluating the utility of HIEs and EHRs for surveillance of chronic diseases, specifically diabetes and hypertension, are limited [6].

Utah has one single HIE that captures health information in all counties and approximately 70% of providers. Since 2009, the Utah Health Information Network (UHIN) has managed this HIE that includes records from 400 clinics and 80 hospitals in Utah, 3 major laboratories, and Utah’s Medicaid medication history [7]. As part of a larger effort to determine the potential to use HIE data for chronic disease surveillance, this analysis evaluated data comparability between original source EHR data to the compiled data in HIE. This was done by assessing demographic and disease-specific (diabetes and hypertension) variables from patients whose records could be found in both a mid-size Utah health system and in the HIE.

## Methods

### Setting

UHIN is a nonprofit coalition of Utah healthcare providers that operates the Clinical Health Information Exchange. This HIE is a voluntary intersystem exchange funded by the health systems that use it. Patient participation in the HIE abides by the Health Insurance Portability and Accountability Act of 1996 [8]. The HIE currently captures a variety of data types from different health systems.

The mid-size Utah health system used in this analysis joined the HIE in 2010. It is an independent accountable care organization made up of approximately 100 outpatient clinics throughout the state with advanced information technology capacity. All providers in this health system send EHR data, collected using Allscripts™ (Chicago, USA), to the systems own data warehouse (Database A) and to the HIE (Database B). These facilities send continuity of care documents (CCD) to their own data warehouse, but only send transcription notes and general laboratory information to the HIE (Database B) (Additional file 1: Figure S1). Because the HIE receives only transcription notes, the HIE data warehouse uses natural language processing to abstract information from those unstructured fields.

Each data warehouse also captures data from other health facilities. Some of these other healthcare facilities share data with both data warehouses and some share with only one data warehouse. Because of this, we did not expect that all encounters would match between the two databases.

### Data source

Patients were defined as any person with at least one encounter in the mid-level health system facility during 2015. Encounters were defined as an EHR message documenting an interaction between a patient and a healthcare practitioner. The Utah Department of Health was able to obtain datasets from both Database A and Database B. Researchers did not have access to the warehouses, but were given flat data files containing: medical record number, date of service, facility of service, patient month and year of birth, sex, race, A1C test date and result, blood pressure reading (systolic and diastolic) and date, and diagnosis codes associated with the encounter (ICD-10-CM and ICD-9-CM). Additionally, Database A contained CPT® (Current Procedural Terminology) codes (American Medical Association, Chicago, Illinois) and a variable for facility names.

### Data cleaning

**Fig. 1:**
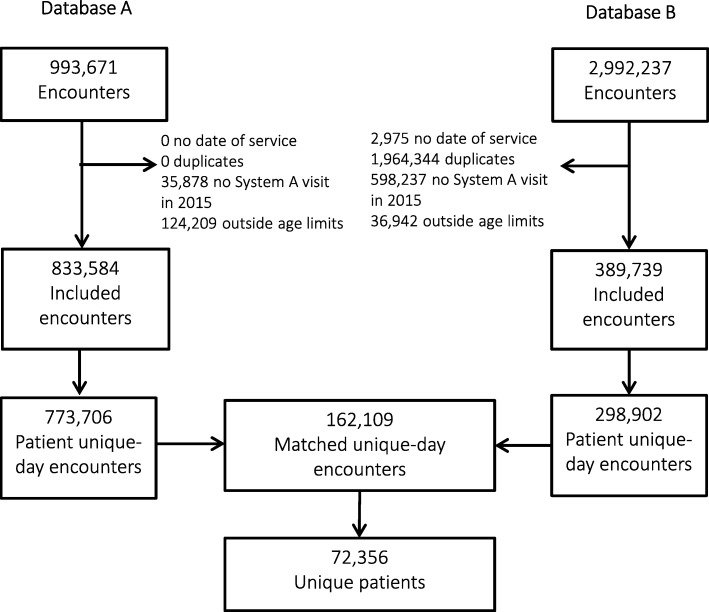
Data flow diagram of a mid-size health system (Database A) and health information exchanges (Database B) encounter-level data matching process, Utah — 2015

We performed deduplication on Database B, which had not been deduplicated by the system prior to our receipt. The following encounters were excluded (Fig. 1): (1) encounters with missing date of service, (2) encounters for patients with < 1 encounter in the mid-level healthcare system in 2015, or (2) encounters from patients aged < 18 years, > 85 years or missing age.

Patient medical record number and date of service were joined to create a unique patient encounter day variable. Information for patients with > 1 encounter on the same day was aggregated for that day. Database A and Database B were matched based on unique patient day encounter and only matching encounters were retained (Fig. 1). Matched unique-day encounters were subsequently aggregated by patient.

### Variable definitions

Variables for diabetes and hypertension were created based on established guidelines [9, 10]. Only a single reading was available for each visit. Implausible A1C results, those equal to 0 or greater than 50, were categorized as missing (*n* = 61). A1C test results for a given encounter were also categorized as missing if the date of the test preceded the encounter date. Implausible blood pressure readings, systolic pressure greater than 260 or less than 50 or diastolic pressure greater than 200 or less than 0, or those dated with any date before the encounter day were reclassified as missing (*n* = 291).

Patients were classified as having diabetes (Additional file 2: Figure S2) if they were aged 18–85 years and had ≥1 A1C reading of ≥6.5% (48 mmol/mol) in 2015 or had diagnosis or administrative codes (ICD-9-CM, ICD-10-CM or CPT®), as listed in the National Quality Forum (NQF) 0059 [9]. Patients were classified as having hypertension based on diagnosis or administrative codes (ICD-9-CM, ICD-10-CM or CPT®) as listed in the NQF 0018 [10]. Patients were classified as having hypertension (Additional file 2: Figure S2) if they were aged 18–79 years and had ≥1 blood pressure reading of ≥140/90 mmHg (or ≥ 130/80 mmHg and a diabetes diagnosis); if they were aged 80–85 years and had ≥1 reading of ≥150/90 mmHg on separate days (or ≥ 140/90 mmHg and a diabetes diagnosis) [11].

### Analysis

In our analysis, we (1) assessed data concordance between sources, (2) estimated sensitivity and positive predictive values for diabetes and hypertension, (3) calculated adjusted prevalence of diabetes and hypertension in Database A and Database B, and (4) compared slopes of disease prevalence over age in both systems.

R version 3.4 was used to conduct descriptive analysis [12]. The ‘data.table’ package in R was used to aggregate data by patient-day encounter and then again by patient [13]. To estimate concordance of estimates between Database A and Database B, we used the most recent test or reading available for each patient. We only considered exact matches to be concordant.

To estimate sensitivity and positive predictive value, we used Database A as the ‘gold standard’ because it was the primary data collector. Sensitivity was defined as the number of persons in both Database A and Database B classified as having the condition (either diabetes or hypertension) divided by the number of persons classified with the condition in Database A alone. Positive predictive value was defined as the number of persons in both Database A and Database B classified with the condition (either diabetes or hypertension) divided by the number of all persons classified with the condition in Database B alone.

The ‘survey’ package in R was used for iterative proportional fitting (raking) using American Community Survey 2015 marginal proportions for sex and 5–year-age groups [14]. We calculated adjusted prevalence estimates for diabetes and hypertension using this weighting procedure. Raked age- and sex-stratified estimates were calculated for diabetes and hypertension. To test whether the slopes of the population adjusted prevalence estimates across age groups were the same between Database A and Database B, we created aggregated data tables of the adjusted prevalence for each system by age group and sex. Linear regression models with prevalence as an outcome and regressors for ordinal age group (1: 18–34, 2: 35–49, 3: 50–64 and 4: 65–85), dummy variables for system, and an age group-by-system interaction term were generated.

The model is represented by:

$$ Yt=\beta 0+\beta 1T+\beta 2 Xt+\beta 3 TXt $$

Y: Prevalence at age group T

T: Age group (treated as an ordinal variable for trend)

X: A dummy variable indicating system

*β*3 indicates the slope change across systems. T-tests were used to test the hypothesis that the slopes were the same and the criterion for cut-off was *P* < 0.05. Separate models were fitted for the total population and for both sexes.

## Results

### Data cleaning

The dataset received from Database A contained 993,671 encounters (Fig. 1). These data were cleaned and aggregated to patient unique-day resulting in 773,706 observations. Database B contained 2,992,237 encounters; cleaning and aggregating resulted in 298,902 observations. After matching Database A and B, there were 162,109 unique patient encounters by 72,356 patients.

In all, 21.0% of Database A and 54.2% of Database B patient unique-day encounters matched. These matched patients were 56.0% female, and 32.4% were aged 65–85 years, 24.8% were aged 50–64 years, 19.8% were 35–49 years, and 23.0% were 18–34 years (not shown). Half (49.7%) had a single health encounter, 20.8% had two encounters, and 29.6% had ≥3 encounters in 2015.

### Data concordance

Concordance of 2015 data between Database A and Database B was 99.8% for month and year of birth and 99.1% for sex (Table 1). The most recent A1C test result values was concordant for 89.8% of patients; 5.1% of patients were missing values in Database A and not Database B, while 4.1% were missing values in Database B and not in Database A. Approximately half (54.2%) of most recent systolic blood pressure readings were concordant between the two systems. Discordant values for data missing in one system and not the other was 30.7% for Database A and 10.0% for Database B. Concordance of race was not assessed because Database B was missing race data for 87.5% of patients.

**Table 1:** Concordance of matched patient-level data from patients in a mid-size health system own data warehouse (Database A) to those patients data in a health information exchange data warehouse (Database B) and), Utah — 2015 (*N* = 72,356)

Variables	Concordant Values^a^	Discordant Values	Missing Value in Database A	Missing Value in Database B
	No. (%)	No. (%)	No. (%)	No. (%)
Month and Year of Birth	72,246 (99.8)	110 (0.2)	0	0
Sex	71,710 (99.1)	646 (0.9)	0	0
A1C Test Result^b^	65,006 (89.8)	707 (1.0)	3701 (5.1)	2942 (4.1)
Systolic Blood Pressure Reading^b^	39,224 (54.2)	3649 (5.1)	22,232 (30.7)	7251 (10.0)
Diastolic Blood Pressure Reading^b^	39,140 (54.1)	3733 (5.2)	22,232 (30.7)	7251 (10.0)

^a^Including concordant values when both datasets have missing values

^b^From most recent result available in 2015

### Disease classification

Sensitivity and positive predictive value were 22.9 and 36.0%, respectively, when using only A1C test result to classify a person as having diabetes (Table 2); this measure captures only individuals with uncontrolled diabetes. When comparing blood pressure readings alone, without diagnostic codes, for classification of hypertension (which only captures persons with uncontrolled hypertension), sensitivity and positive predictive value were 16.5 and 54.4%, respectively. Classification based on both clinical readings and diagnostic codes performed better (approximately 50% for specificity and 70% for positive predictive value for both diabetes and hypertension) than clinical readings alone.

**Table 2:** Sensitivity, positive predictive value, and proportion of patients classified as having diabetes and hypertension by different methods, using matched patient-level data in a mid-size health system (Database A) to those systems patients in the Health Information Exchange (Database B), Utah — 2015 (*N* = 72,356)

Variables	Sensitivity^a^	Positive Predictive Value^a^	Proportion Classified in Database A	Proportion Classified in Database B	Absolute Difference	Percent Difference
	%	%	%	%	%	%
Classified as having Diabetes based on:						
A1C Test Results^b^	22.9	36.0	1.2	0.8	0.4	40.0
Diagnosis Codes	52.8	76.9	11.6	8.0	3.6	36.7
Both^c^	55.7	77.8	11.6	8.3	3.3	33.2
Classified as having Hypertension based on:						
Blood Pressure Readings^b^	16.5	54.4	3.0	0.9	2.1	107.7
Diagnosis Codes	57.4	70.6	20.7	16.8	3.9	20.8
Both^c^	57.1	71.4	21.5	17.2	4.3	22.2

^a^Using Database A as the standard

^b^Only captures persons with either uncontrolled diabetes or uncontrolled hypertension

^c^Used as measure of disease prevalence

### Disease prevalence

The 2015 population-adjusted prevalence of diabetes and hypertension differed by more than 3% between data sources for hypertension and diabetes (8.4% in Database A vs 5.8% in Database B for hypertension and 14.5% in Database A vs 11.6% in Database B) (Table 3). However, the slope of age-stratified prevalence across age groups did not differ between systems for either condition. When stratified by both sex and age, the slope of prevalence did differ for males for both conditions.

**Table 3:** Crude and adjusted prevalence of diabetes and hypertension in a mid-size health system (Database A) to those systems patients in the Health Information Exchange (Database B), Utah — 2015 (*N* = 72,356)

	Age group	Database A			Database B			
		n	Crude %	Adj^a^ %	n	Crude %	Adj^a^ %	*P*-value^b^
Diabetes								
Male	18–34	6370	2.7	2.6	6360	1.5	1.5	0.09
	35–49	6207	7.5	7.3	6193	4.8	4.6	
	50–64	8228	14.6	14.3	8188	10.5	10.3	
	65–85	10,898	22.4	22.1	10,810	16.6	16.2	
Female	18–34	11,028	3.8	3.7	10,268	2.1	2.1	0.40
	35–49	8106	5.8	5.7	8083	3.9	3.8	
	50–64	9700	11.1	10.8	9646	8.3	8.0	
	65–85	12,442	17.3	17.2	12,307	13.2	13.1	
All	18–34	16,657	3.3	3.2	16,661	1.9	1.8	0.21
	35–49	14,334	6.6	6.5	14,334	4.2	4.2	
	50–64	17,955	12.7	12.7	17,954	9.3	9.3	
	65–85	23,410	19.7	19.9	23,406	14.6	14.8	
	18–85	72,356	11.6	8.4	72,356	8.3	5.8	
Hypertension								
Male	18–34	6370	4.9	4.9	6360	2.7	2.7	0.03
	35–49	6207	14.1	13.8	6193	11.9	11.6	
	50–64	8228	28.1	27.7	8188	22.2	21.7	
	65–85	10,898	42.3	41.6	10,810	31.8	31.2	
Female	18–34	11,028	2.5	2.5	10,268	1.4	1.4	0.60
	35–49	8106	8.0	7.8	8083	7.0	6.8	
	50–64	9700	18.6	18.1	9646	17.1	16.6	
	65–85	12,442	37.9	36.9	12,307	32.0	31.1	
All	18–34	16,657	3.5	3.6	16,661	1.9	2.0	0.24
	35–49	14,334	10.7	10.9	14,334	9.1	9.2	
	50–64	17,955	23.0	23.2	17,954	19.3	19.3	
	65–85	23,410	40.0	39.4	23,406	31.5	31.2	
	18–85	72,356	21.5	14.5	72,356	17.2	11.6	

^a^Adjusted by iterative proportional fitting (raking) using American Community Survey 2015 marginal proportions for sex and 5 year age groups

^b^T-test to compare the slope of Database A prevalence across age groups to the slope of Database B prevalence across age groups

## Discussion

This study found important quality gaps in the use of clinical data for surveillance of diabetes and hypertension at a population level; nonetheless, high concordance of structured data demonstrate promise in an HIEs capacity to adequately capture data. When comparing age and sex, both structured data elements, for patients in Database A to matched data for those patients in the HIE (Database B), our analysis revealed approximately 99% were the same. In addition, while prevalence of disease was not the same in both health systems, this difference was consistent across age groups as demonstrated by parallel slopes of prevalence over age groups.

Nonetheless, this analysis reveals several gaps in data reliability, especially for hypertension. Only half of blood pressure readings were concordant between the two systems. High discordance of values for blood pressure readings, primarily from discordancy of missing values, resulted in high misclassification of hypertension. Sensitivity and positive predictive values based on blood pressure readings alone were only 16.4 and 54.4%, respectively. A possible explanation for this discordance is the way in which the mid-level health system sends data to the HIE. Data is sent using unstructured transcript notes, which the HIE reads using natural language processing, but the health system sends structured data to its own data warehouse. Natural language processing was able to detect additional information that the health system’s own data warehouse was not getting in their structured fields, conversely the HIE was not getting data from the structured fields.

Other studies have reported similar concerns with concordance between EHR systems and HIE data warehouses [15, 16]. While transcript data is a preferable format for an HIE’s primary function of enabling practitioners to follow patients across health systems, our analysis shows that it functions poorly for surveillance purposes. Data integrity might improve by requiring health systems to send patient data in structured formats to the HIE, such as through CCD [17].

The inability for the HIE to consistently capture important demographic information (e.g., race) and socioeconomic variables from patients was another gap identified in the HIE’s readiness to function as a statewide chronic disease surveillance system. Reliable data collection on race, ethnicity, and language by EHRs is difficult [18]. High rates of misclassification and missing information have been documented across studies [19, 20], even in settings with regulations promoting collection of these data [21]. This limitation could be mitigated by requiring health systems to capture and report structured codes for race and similar demographic and socioeconomic characteristics to the HIE.

Some limitations exist in this analysis. First, the prevalence estimates of diabetes and hypertension are specific to the population studied (i.e., patients in a mid-level health system in Utah in 2015), and these estimates cannot be extrapolated to other populations. The results presented in this analysis are not representative of the entire Utah healthcare seeking population nor do they capture non-health seeking populations. Similarly, only a single health system was analyzed, and we do not know if these issues persist across health systems. In order to fully understand the utility of the HIE for surveillance, additional analysis on other health system that share data with the HIE would be necessary. Secondly, disease classification based on 1 year of healthcare encounters will fail to capture persons with disease who had just one encounter for an unrelated health event. For example, a patient having high blood pressure in a single visit will not have been classified as having hypertension, even if they may have had two high blood pressure readings the previous year. Though this prevents over estimation of hypertension from when blood pressure temporarily increases with illness, it might underestimate hypertension in persons with only a single health encounter. Lastly, our analysis does not differentiate between data quality problems resulting from data entry errors and data transformation errors.

## Conclusion

In conclusion, this study found that the Utah HIE is capable of providing useful, although limited, information for surveillance of diabetes and hypertension. Given its potential, a greater understanding is needed of the mechanisms by which HIEs capture, process, and store EHR data from multiple health systems, and how these processes affect measures of diabetes and hypertension. Public health agencies in places with fragmented healthcare and EHR systems, like Utah, might consider working with HIEs to address data quality issues, such as by mandating use of structured data fields, so that EHR data can be harnessed for population level chronic disease surveillance.

## Additional files

Additional file 1: **Figure S1.** Data flow diagram of patient data across healthcare systems into data warehouses A and B, Utah — 2015. (PDF 430 kb)

Additional file 2: **Figure S2.** Diabetes and hypertension classification based on available electronic health record data, Utah — 2015. (PDF 394 kb)

## Data Availability

The data that support the findings of this study are available from the Utah Health Information Network but restrictions apply to the availability of these data, which were used under agreement for the current study, and so are not publicly available.

## Abbreviations

EHR: Electronic Health Record
HIE: Health Information Exchange
