# Supplementary figures and images for: Use of electronic health records from a statewide health information exchange to support public health surveillance of diabetes and hypertension
Source: BMC Public Health. 2019 Aug 14;19:1106. doi: 10.1186/s12889-019-7367-z (PMC6694493; doi:10.1186/s12889-019-7367-z)

**Diabetes Classification**

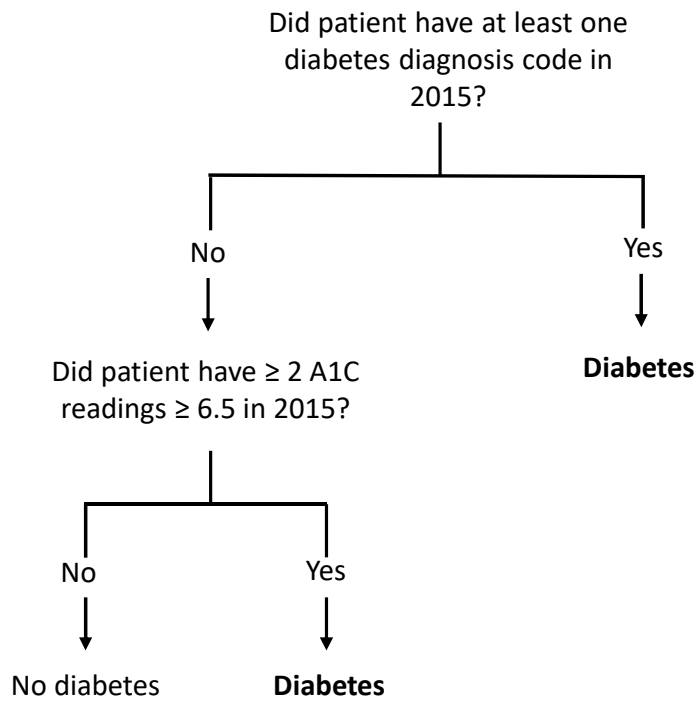

**Hypertension Classification**

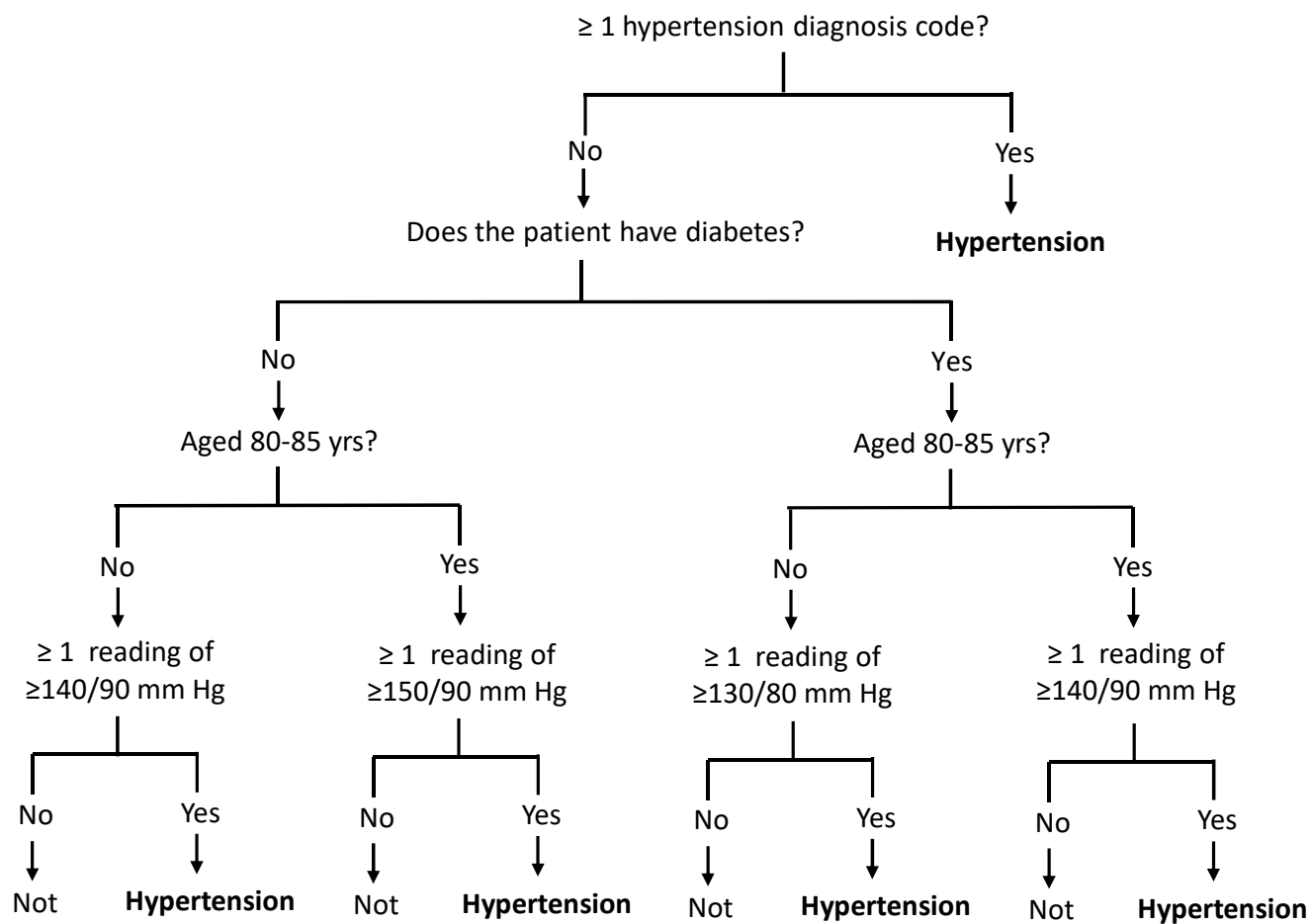

Supplement: Supplementary file 2 — Figure S2. Diabetes and hypertension classification based on available electronic health record data, Utah — 2015. (PDF 394 kb) [file 12889_2019_7367_MOESM2_ESM.pdf]
