# Supplementary material for: Use of electronic health records from a statewide health information exchange to support public health surveillance of diabetes and hypertension
Source: BMC Public Health. 2019 Aug 14;19:1106. doi: 10.1186/s12889-019-7367-z (PMC6694493; doi:10.1186/s12889-019-7367-z)
Supplement: Supplementary file 1 — Figure S1. Data flow diagram of patient data across healthcare systems into data warehouses A and B, Utah — 2015. (PDF 430 kb) [file 12889_2019_7367_MOESM1_ESM.pdf]

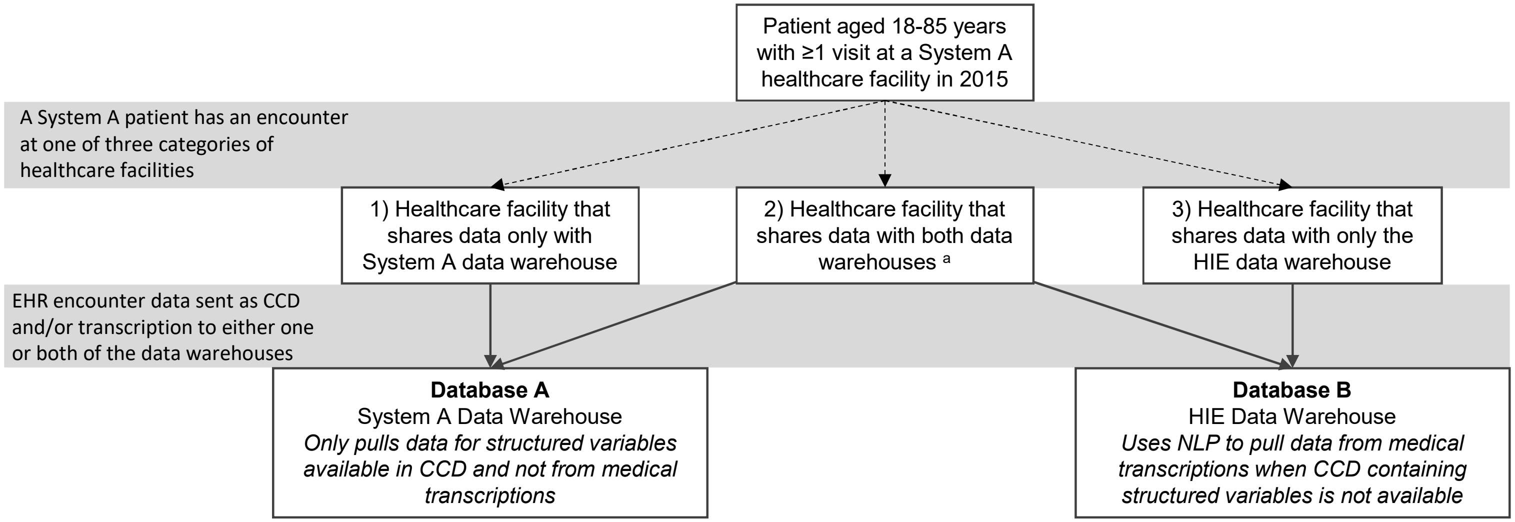


[a] All Healthcare System A facilities are under category 2, but each data warehouse receives data from System A patients from a variety of healthcare systems and healthcare facilities.

CCD: Continuity of care document, EHR: Electronic health record, HIE: Health information exchange, NLP: Natural language processing,
